# Etiology, Severity, Audiogram Type, and Device Usage in Patients with Unilateral Moderate to Profound Sensorineural Hearing Loss in Japan

**DOI:** 10.3390/jcm12134290

**Published:** 2023-06-26

**Authors:** Hajime Koyama, Akinori Kashio, Shinichi Nishimura, Haruo Takahashi, Shinichi Iwasaki, Katsumi Doi, Takashi Nakagawa, Ken Ito, Tatsuya Yamasoba

**Affiliations:** 1 Department of Otolaryngology and Head and Neck Surgery, Graduate School of Medicine, The University of Tokyo, Tokyo 113-8654, Japan; hakoyama-tky@umin.ac.jp (H.K.); kashioa@gmail.com (A.K.); snishimura-tky@umin.ac.jp (S.N.); 2 Department of Otolaryngology, Mitsui Memorial Hospital, Tokyo 101-8643, Japan; 3 Department of Otolaryngology, Nagasaki Harbor Medical Center, Nagasaki 850-0842, Japan; htak0831@nagasaki-u.ac.jp; 4 Department of Otolaryngology and Head and Neck Surgery, Nagoya City University Graduate School of Medical Sciences, Nagoya 467-8601, Japan; iwasaki0824@gmail.com; 5 Department of Otolaryngology, Faculty of Medicine, Kindai University, Osaka 589-0014, Japan; kdoi@mopera.net; 6 Department of Otorhinolaryngology, Graduate School of Medical Sciences, Kyushu University, Fukuoka 812-8582, Japan; nakagagwa.takashi.284@m.kyushu-u.ac.jp; 7 Department of Otolaryngology, Faculty of Medicine, Teikyo University, Tokyo 173-8605, Japan; itoken-tky@umin.ac.jp

**Keywords:** hearing aid, cochlear implant, unilateral sensorineural hearing loss

## Abstract

Background: Few studies have reported on the etiology, severity, or device usage of unilateral sensorineural hearing loss (UHL) compared to bilateral hearing loss. Therefore, this study investigated the characteristics of UHL in adults and children. Methods: We performed a survey using questionnaires for secondary and tertiary otolaryngology institutions. Results: We included 15,981 patients (1549 children and 14,432 adults) from 196 institutions with otolaryngology residency programs and 2844 patients (336 children and 2508 adults) from 27 institutions with board members of the Japan Audiology Society. The latter submitted audiological data. Among children, most diagnoses were made at age 0. Approximately half of them had profound hearing loss, and 37 children (2.2%) used hearing devices. Among adults, the number of cases increased with age, but decreased when people reached their 80s and 90s. More than half of them had moderate hearing loss. Sudden sensorineural hearing loss was the most common cause of UHL of all ages; 4.4% of UHL patients used hearing devices, and most of the device users (98.6%) selected a conventional hearing aid. Conclusions: Hearing aid use is limited in children and adults with UHL in Japan. There could be many candidates with UHL for intervention such as a cochlear implant.

## 1. Introduction

Humans have two ears so that they can communicate with each other in difficult situations effectively. Binaural hearing enables good coordination between the two ears, giving the benefit of the binaural summation effect, the squelch effect, and the head shadow effect. Binaural summation results in binaural redundancy and benefit beyond increased loudness [1,2,3]. The squelch effect enables the brain to suppress noise by the contralateral noise information. The head shadow effect makes the binaural listeners attend to the ear with a better sound-to-noise ratio especially when speech and noise are spatially separated [4]. Binaural hearing also creates interaural time or intensity differences, which enables it to detect sound sources [5,6].

On the other hand, unilateral sensorineural hearing loss (UHL), a condition where hearing is normal in one ear but is impaired in the other ear, has fewer advantages than binaural hearing and several negative effects on hearing in both children [7,8,9] and adults [10]. Children with UHL have difficulty in sound localization and less accurate spatial hearing [11,12,13]. A report suggested that children with UHL achieved a mean localization error range of 28 degrees, while children with normal hearing achieved 4 to 6 degrees [14,15]. Children with UHL also showed poorer performance in speech perception in noisy conditions [16,17], especially when the noise was moved to the impaired ear [14]. In addition, children with UHL show failure in school tests, poorer language development [18], and lower IQ compared to children with normal hearing [19]. This is because they need better speech perception in noise than adults for learning [20], as young children spend a lot of time in noisy environments such as school [21], and this is not being achieved.

Adults with UHL as well have less ability to localize sounds and awareness of incoming sounds and understand speech in noise [22]. This leads to significant difficulties with hearing in most daily activities, resulting in adult patients with UHL experiencing difficulty and frustration with speech communication [23], and UHL can lead to reduced self-confidence, embarrassment, and withdrawal from social contact [24]. Therefore, the need to manage UHL is important.

Recently, several options such as hearing aids with the contralateral routing of signals (CROS) [25,26], bone conductive devices [27,28], and cochlear implants (CI) [29,30] have been reported as treatments for UHL. A former article reported the benefit of the options could be influenced by the age of onset and etiology of UHL [31]. Therefore, etiological and audiological data are important to assess the candidacy of these options for UHL patients.

Despite its importance, there are only a few reports on etiological and audiological data for UHL compared to bilateral hearing loss. In Japan, there are no guidelines or recommendations as to other treatment modalities for UHL, such as CROS hearing aid or bone-anchored devices, and CI for UHL is not approved by the national insurance system. Information on the incidence, etiology, severity, and device usage of UHL is essential for considering the options for UHL. Therefore, we investigated these parameters in adults and children with UHL in Japan to identify patients who are far from hearing device intervention.

## 2. Materials and Methods

We conducted a questionnaire survey of institutions with otolaryngology residency programs including institutions with board members of the Japan Audiological Society (JAS) (Table S1). The medical records of the patients’ first visits were reviewed over 2 years (April 2018 to March 2020) for the former institutions and over 3 years (April 2017 to March 2020) for the latter institutions. Age, gender, etiology, severity, and intervention were collected from former institutions, and age, gender, etiology, pure tone audiogram data, and intervention were also corrected from the former institution. Etiologies of the patients were determined by the providers at the institutions. Only one etiology was selected from acquired diseases (sudden sensorineural hearing loss, auditory tumor, Meniere’s disease, perilymphatic fistula, acoustic trauma, other trauma, mumps infection, meningitis, and other detected causes or unknown causes) or congenital diseases (inner ear anomaly, cochlear nerve canal stenosis, cytomegalovirus infection, other infection, and other detected causes or unknown causes). If patients received an intervention, the type of intervention was selected from bone-anchored hearing aids, hearing aids (air, bone or cartilage conductive hearing aids or contralateral routing system) or cochlear implants. This study was approved by the review board of our institution (2020191NI).

### 2.1. Patients

UHL was defined as moderate, severe or profound hearing loss (≥40 dB HL) in one ear and normal hearing (<20 dB HL) in the other ear [32]. Patients with mild hearing loss (≥20 dB HL and <40dB HL) were excluded from this study. One reason for this is that this survey was conducted at secondary or tertiary institutions, which are not often visited by patients with mild hearing loss in Japan, and therefore the causes of mild hearing loss at these institutions are likely not to reflect the actual situation. Another reason is to increase the collection rate of the questionnaire by reducing the number of cases to be reported. Information on age, sex, etiology, severity of hearing loss, and hearing device use of patients with UHL was collected. From only the institutions with JAS board members, the thresholds of the pure tone audiogram, auditory brainstem response (ABR), and/or auditory steady-state response (ASSR) were also collected. We calculated the average hearing threshold by using 3 frequencies (0.5 kHz, 1 kHz, and 2 kHz) and categorized the patients’ severity as moderate (average hearing threshold of 40 dB to less than 70 dB), severe (average hearing threshold of 70 dB to less than 90 dB) or profound (average threshold of 90 dB or more). However, audiograms (or ABR/ASSR) were not provided to us from the secondary or tertiary institutions; the categorization of their severity was performed by the otolaryngologists who worked in each institution based upon the patients’ audiograms (or ABR/ASSR) according to the same criteria. Patients were divided into two groups according to their age as follows: children (<18 years) and adults (≥18 years).

### 2.2. Definitions

When the severity of hearing loss was not filled in, the severity of the patient’s hearing was defined as unknown. The ABR threshold or average ASSR was used as a substitute for pure-tone average in children who could not be assessed with pure-tone audiograms.

For the subset collected from the institutions with JAS board members, hearing loss was also categorized into low-tone and high-tone hearing loss. Low-frequency ascending (LFA) hearing loss was defined as when the low-tone threshold (average of 125, 250, and 500 Hz) was 20 dB worse than the high-tone threshold (average of 2000, 4000, and 8000 Hz). High-frequency sloping (HFS) loss was defined as when the high-tone threshold was 20 dB worse than the low-tone threshold.

## 3. Results

We sent the questionnaires to 592 institutions that have the otolaryngology residency program and 31 institutions that JAS board members belong to. Completed questionnaires were obtained from 196 of 592 institutions in the otolaryngology residency program and from 27 of 31 institutions with JAS board members. We analyzed data of 15,981 patients (1549 children and 14,432 adults) from the former institutions and 2844 patients (336 children and 2508 adults) with audiogram data from the latter institutions. 

### 3.1. UHL in Children

**Figure 1:**
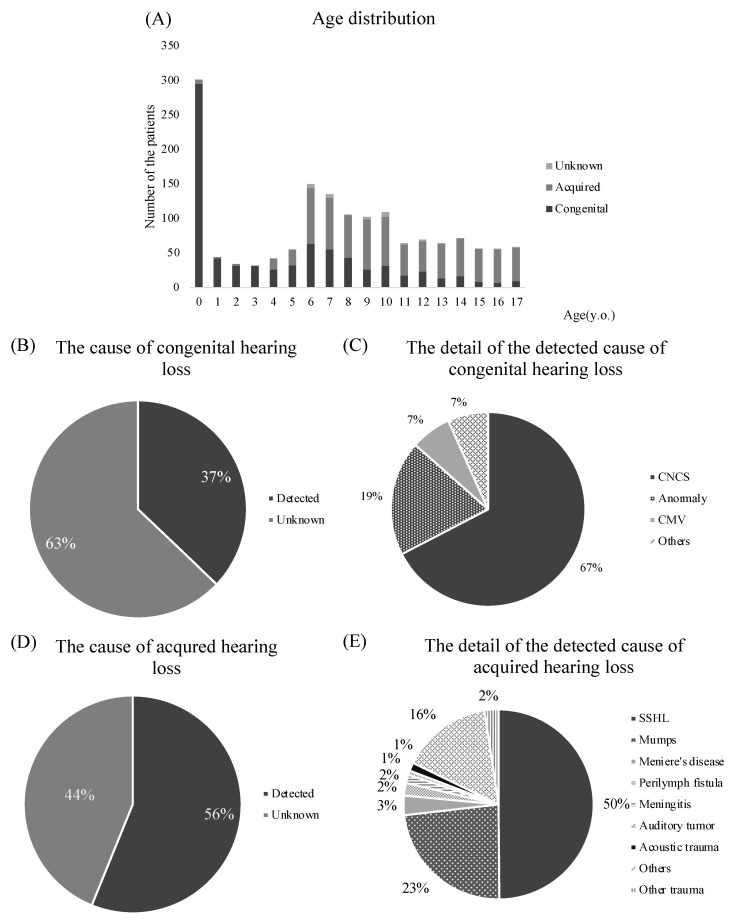
Age distribution of children with unilateral hearing loss (**A**), causes of congenital (**B**) and acquired hearing loss (**D**), and detailed causes of congenital and acquired hearing loss (**C**,**E**) in children. CNCS, cochlear nerve canal stenosis; CMV, cytomegalovirus; SSHL, sudden sensorineural hearing loss.

Figure 1A shows the distribution of age at referrals for children with UHL. The most common age at referral was 0 years. The number of children referred as potentially UHL increased between the ages of 6 and 7 years, and 26.1% of UHL children were considered to have etiologically congenital UHL after age 7 years. Only 1.5% of children with acquired UHL were referred at age 3 or younger. The number of referred children with acquired hearing loss increased from the age of 4 years to 6 years (16, 22, and 81, respectively) and stabilized at the age of 6 years and older (44 to 81; mean, 57.3). The breakdown is as follows: inner ear anomaly was the most frequent cause among UHL children at the age of 3 and 4 years (9.5% and 9.7%, respectively); 86.8% of cochlear nerve canal stenosis (CNCS) UHL children were referred at 9 years old or younger and 58.4% of CNCS UHL children were diagnosed at 5 years old or younger; 98.0% of UHL children due to mumps were detected at age 4 or older. SSHL became more common with increasing age (0.0–4.8%, 4.7–23.4%, 26.1–44.6% of UHL children between 0–5 years old, 6–11 years old, and 12–17 years old, respectively).

Figure 1B–D shows the etiologies of congenital and acquired hearing loss in children. Among children considered as experiencing congenital hearing loss, the cause was unknown in 63% (*n* = 483) of cases (Figure 1B), and in the known causes, CNCS was the most common (Figure 1C). 

Fifty-six percent of the causes were detectable in pediatric cases of acquired hearing loss (Figure 1D), and sudden sensorineural hearing loss (SSHL) was the most common cause, followed by mumps and Ménière’s disease (Figure 1E). 

**Figure 2:**
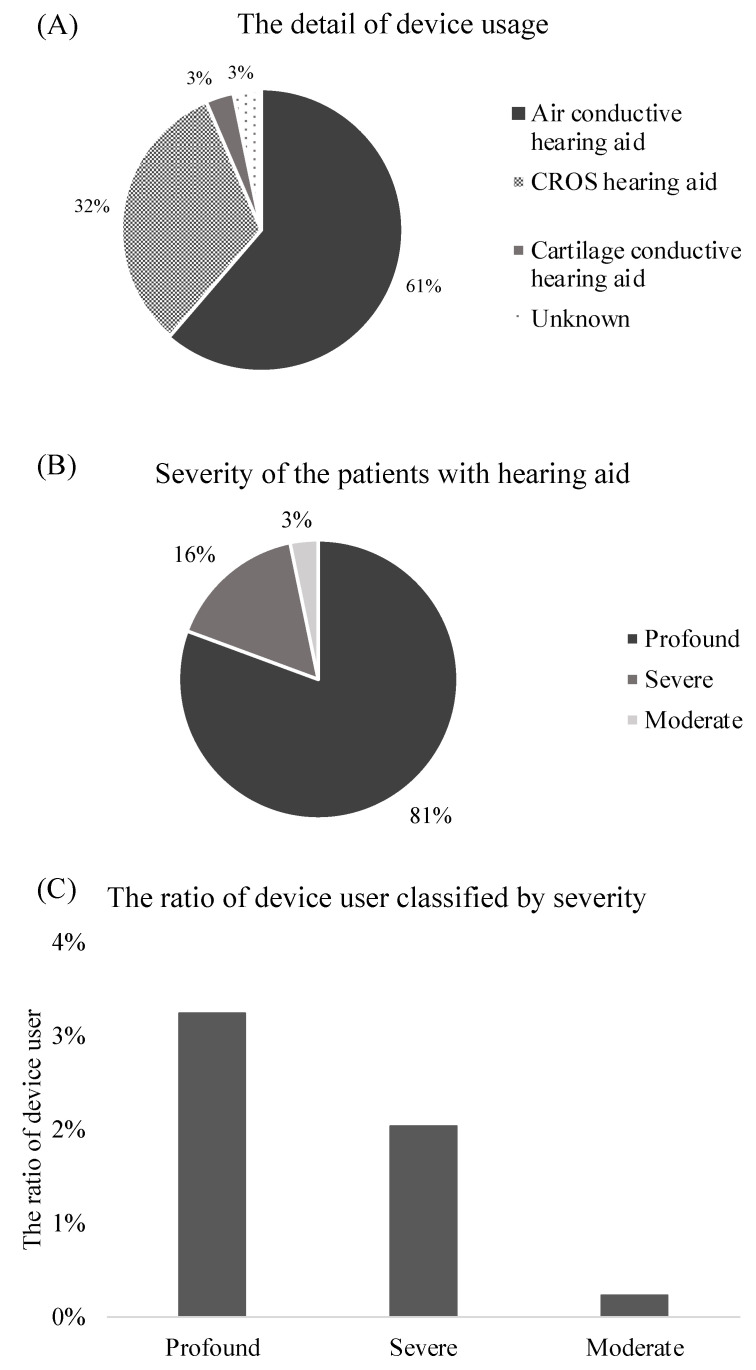
Hearing device usage in children. Type of hearing device (**A**), hearing loss severity among those with hearing aids (**B**), and percentage of patients with hearing aid for each category of severity (**C**). CROS, contralateral routing system.

In terms of severity, about half of the patients with both congenital and acquired hearing loss had profound hearing loss, except that all patients with hearing loss due to mumps had profound hearing loss. Hearing devices were used in 31 patients (2.2%), of which air-conductive hearing aids were used in 19 (62%), followed by CROS hearing aids in 10 (32%) (Figure 2A). In these children, profound hearing loss was the most common (*n* = 25, 81%), followed by severe (*n* = 5, 16%) and moderate (*n* = 1, 3.2%) hearing loss (Figure 2B). The ratios of devices used for patients with profound, severe, and moderate hearing loss were 3.2%, 2.0%, and 0.23%, respectively. There was a tendency that children with profound UHL to use hearing aids more frequently than children with moderate UHL.

### 3.2. UHL in Adults

**Figure 3:**
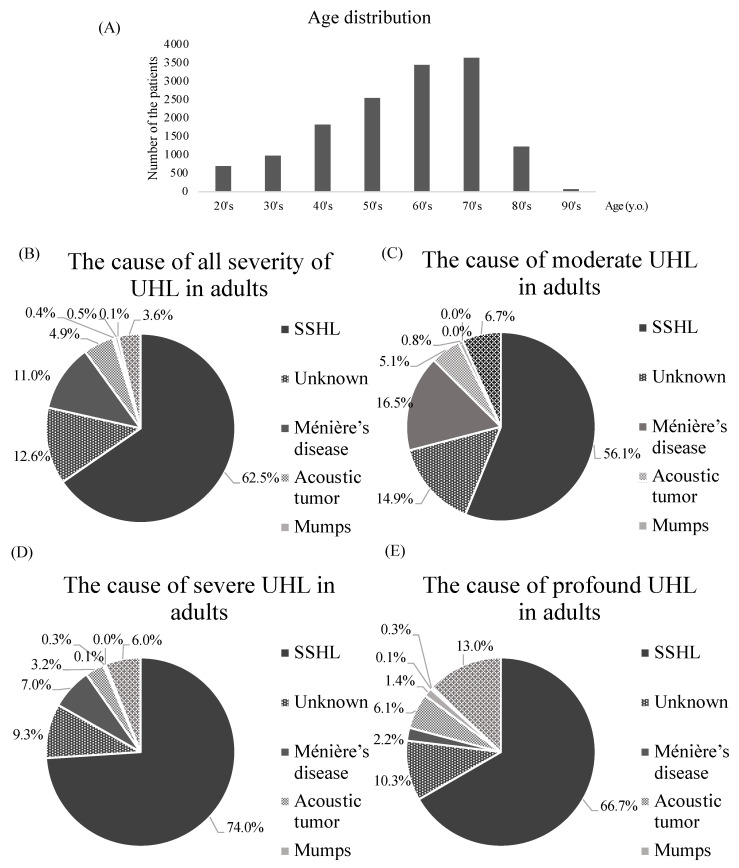
Age distribution of adults with unilateral hearing loss (**A**) and the causes of UHL for each severity (**B**–**E**). SSHL, sudden sensorineural hearing loss; UHL, unilateral hearing loss; y.o., years old.

Figure 3 shows the number of adult cases in each age group by decade (A), the percentages of diseases in all cases (B), and each severity (C–E). The number of patients increased with age until their 70s (20s or younger, *n* = 694; 30s, *n* = 981; 40s, *n* = 1823; 50s, *n* = 2546; 60s, *n* = 3443; 70s, *n* = 3637) and decreased in the 80s and 90s (80s, *n* = 1231; 90s, *n* = 77). SSHL was the most common cause of UHL of all ages (49–66%), followed by unknown causes (8.6–33%) and Ménière’s disease (5.2–13%). Ménière’s disease was more frequent between the ages of 40 and 70 than other ages (11–13% vs. 5.2–9.3%), acoustic tumor was more frequent between the ages of 40 and 60 (5.7–6.7% vs. 2.3–4.1%), and mumps and acoustic trauma were more frequent in the 20s and 30s (1.2–2.7% vs. 0.0–0.5% and 1.1–2.3% vs. 0.0–0.4%, respectively).

In severity, more than half of the patients (55%, *n* = 7904) had moderate hearing loss. Severe and profound hearing loss accounted for almost half of the remainder (22%, *n* = 3155 and 24%, *n* = 3457, respectively). SSHL was a major cause of UHL in all types of severity (moderate: *n* = 4435; 56%, severe: *n* = 2334, 74%; profound: *n* = 2305, 67%). The rate of Ménière’s disease decreased with increased severity (moderate: *n* = 1301, 17%; severe: *n* = 222, 7.0%; profound: *n* = 76, 2.2%).

In the type of audiogram, the horizontal (flat) type of hearing loss was the most common (*n* = 1633, 65%), followed by HFS type (*n* = 702, 28%) and LFA type (*n* = 173, 6.9%). Regarding acoustic tumors, there was a higher percentage of HFS type than LFA type (*n* = 90, 40% and *n* = 9, 4.0%, respectively), but in Ménière’s disease, the percentage was similar (*n* = 49, 14% and *n* = 48, 14%, respectively).

**Figure 4:**
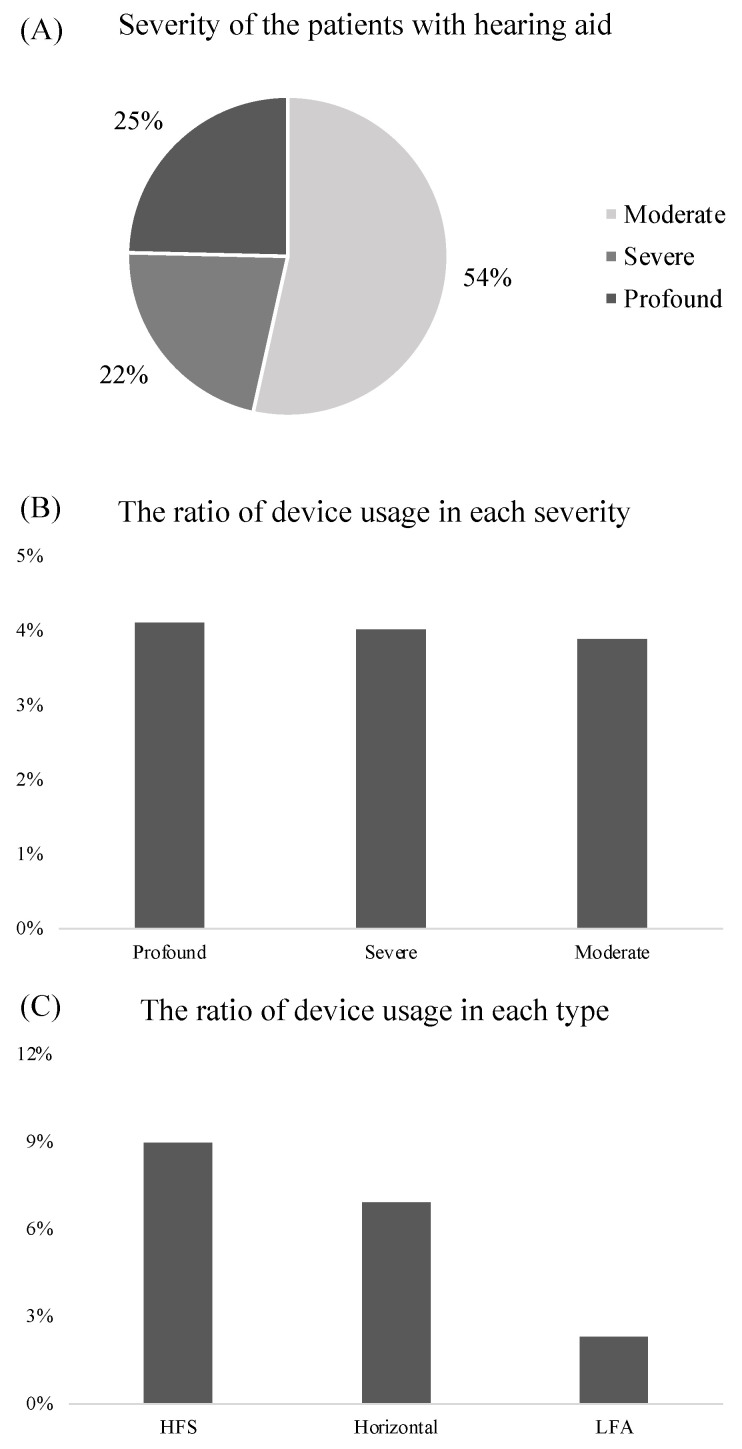
Percentages of the type of severity among adults with hearing devices (**A**), percentages of device use for each type of severity (**B**), and hearing loss pattern (**C**). HFS, high-frequency sloping; LFA, low frequency ascending.

A hearing device (air conductive hearing aid, CROS hearing aid, bone conductive hearing aid, cartilage hearing aid, bone anchored hearing aid, or CI) was used in 581 patients (4.0%), in which most (*n* = 573, 98.6%) used a hearing aid (air conductive hearing aid: *n* = 534, 91.9%; CROS hearing aid: *n* = 26, 4.5%; bone conductive hearing aid: *n* = 2, 0.3%; or cartilage hearing aid: *n* = 1, 0.2%). Moderate hearing loss was predominant (*n* = 308, 54%) among hearing aid users, followed by profound (*n* = 142, 25%) and severe (*n* = 126, 22%) hearing loss (Figure 4A), but there was no clear trend in the proportion of devices usage among the type of severity (Figure 4B). The ratio of hearing aid use in HFS, horizontal, and LFA hearing loss was 9.0% (*n* = 63), 6.9% (*n* = 113), and 2.3% (*n* = 4), respectively (Figure 4C). Patients with HFS hearing loss tended to use hearing aids more frequently than patients with LFA hearing loss.

## 4. Discussion

In this study, we investigated the etiology, severity, audiological type, and device usage in children and adults with UHL in Japan. This study was based on data acquired from institutions nationwide. This is the first national survey of UHL in Japan conducted in a large population with UHL.

UHL was mostly diagnosed at age 0 years in children. This indicates the importance and effectiveness of newborn hearing screening [33,34,35]. Moreover, the age of 6 years was the second most common age at diagnosis, which reflects the importance and effectiveness of preschool medical examinations performed for 6-year-old children in Japan. However, although Japan conducts health examinations for 18-month-old and 3-year-old children, UHL was not detected at the age of 3 years. This suggests that the health examination for 3-year-olds could not detect all UHL children.

Poor detection of UHL in health examinations at the age of 18 months or 3 years could derive from the methods of examination. In a preschool examination, pure tone audiometry is conducted to evaluate the hearing level on the right and left, separately. However, in the health examinations for 18-month-old and 3-year-old children, reactions to sounds are only assessed using the parents’ whispering, and this examination cannot evaluate unilateral hearing. This leads to a delay in identifying UHL until their hearing can be individually assessed in a preschool examination. The development of new screening programs that enable the evaluation of hearing on each side will be needed for these ages.

As to the age distribution of each disease, cases of inner ear anomaly were found at the age of 3 and 4 years, but some patients with anomalies were detected at ages > 7 years. This may be the result of progressive hearing loss due to the anomaly [36]. Mumps was a common cause of UHL after age four. Mumps infection usually occurs during school age [37], and our results support this finding. In our study, SSHL became more common with increasing age; this corresponds to previous reports, which showed that the prevalence of SSHL increased with age (1.2% <9 years old [38]; 3.5% <14 years old [39]; and 6.6% <18 years old [40]).

The etiology of UHL could be established in 37% and 59% of children considered to experience congenital and acquired hearing loss, respectively. Computed tomography (CT) may have been performed less frequently, especially in children, to avoid radiation exposure. This factor could have underestimated the presence of cochlear malformation or cochlear nerve deficiency. Previous studies reported that the etiology of UHL in children could not be identified in 40% [41] to 65% [42] of cases, which corresponds with our result. A previous study reported that some children with UHL had genetic mutations [43]. In our study, genetic evaluation was not performed, which may have decreased the probability of identifying the etiologies of congenital hearing loss.

In children considered to experience congenital hearing loss, CNCS was the most common cause, which corresponds to findings in previous reports [43,44,45,46]. CNCS is considered to reflect cochlear nerve absence and has been traditionally described as a contraindication to CI [47]. It should be noted that CNCS does not always result in profound hearing loss. Other studies also reported that the average hearing level was 87.9 ± 20.0 dB [48] or 75.6 ± 21.0 dB [49], suggesting that there is variety in hearing loss severity due to CNCS. SSHL was the most common type of acquired hearing loss. However, acute sensorineural hearing loss is rarer in children than in adults [40] and is more often associated with laboratory findings such as viral infection [50], compared to SSHL which is still the major cause of acute hearing loss similar to that of adults [51]. Our results also suggest the same conclusion.

In adults, cases increased with age until the 70s, but decreased in the 80s and 90s. This may come from the effect of age-related hearing loss in the better ear. The distribution of severity in adults differed from that in children. Most adults with UHL had moderate hearing loss compared with children, who had severe to profound hearing loss in >60% of cases. This may come from the difference in etiology as SSHL was the major cause of UHL in adults, and 70% of patients with sudden hearing loss achieved a recovery of average pure tone audiogram being better than 60 dB HL [52]. Studies indicate that the severity of SSHL in adults is similar to that in children [38,53], and our data suggest that only 30% of SSHL cases result in profound hearing loss in children. However, unlike adult cases, the rate of moderate hearing loss in children did not increase because of the low proportion of SSHL.

Ménière’s disease was the major cause of UHL in the 40s and 70s, and mumps or acoustic trauma was the major cause in the 20s and 30s. Our results are consistent with the reported etiology of these diseases [36,54,55,56,57].

In our study, the severity of hearing loss did not differ with regard to the use of a hearing aid. In previous investigations of bilateral hearing loss, severity or hearing difficulty was a major factor determining the use of a hearing aid [58,59]. However, a recent study reported that hearing difficulty is not a significant determinant of hearing aid use in UHL [60]. We also found that severity was not a determinant of hearing aid use in adults, indicating differences between bilateral hearing loss and UHL.

Hearing aids were reported to be beneficial for UHL patients [61], but our survey found that the prevalence of hearing aid use was low. The prevalence has been reported to be 2.0% in the United States [62] and 1.56% in South Korea [60]. This low prevalence may be due to less awareness of the disability of UHL than bilateral hearing loss and the lack of financial support. European countries such as the United Kingdom, France, Denmark, and the Netherlands have public healthcare systems covering the hearing aid expense [63]. In the United States, hearing aid usage proportion has been increasing among the population with redeeming hearing aid expenditures such as the Department of Veterans Affairs or low-cost Internet sales [64]. However, other studies have suggested that complex and multifactorial causes lay in the low percentage of hearing aid use [65,66,67], and considering that there have been few surveys conducted on hearing aid use in UHL, more studies are needed to elucidate the influential factors for hearing aid use in UHL patients.

Although there are no guidelines regarding the treatment of UHL in children, there are several options including amplification systems such as conventional hearing aids, CROS hearing aids, and surgical approaches such as bone-anchored hearing aid systems and CI [68]. Previous studies have suggested that a hearing aid could improve the ability to localize sound in younger children (6–9 years old) [69] and improve the quality of life for children with mild-to-moderate UHL [70]. Another study reported that 37% of UHL children acquired hearing aids after appropriate recommendations [71]. Studies indicate that the severity of hearing loss is not associated with hearing aid recommendations in children with UHL [71,72]. Moreover, recent studies also demonstrate that children with severe-to-profound UHL benefit from CI not only in word recognition scores in the CI-only condition [73,74,75] but also in sound localization [31,76,77], speech perception in noisy environments [78,79,80], and linguistic skills [81]. Therefore, CI has been accepted as a treatment for severe to profound UHL in other countries but is not approved in Japan. The current study suggests that many patients with severe to profound UHL remain untreated in Japan and that these individuals could benefit from interventions such as CI. Given that CNCS and severe cochlear anomaly occupy a certain portion of the etiology of UHL in children, however, indications of CI for children with UHL should be considered carefully, because CI would not be appropriate for children with absent cochlear verve or longer history of severe to profound hearing loss in UHL due to maturation of auditory system or auditory cortex [82].

Our study had several limitations. It was based on questionnaires provided to secondary and tertiary medical institutions, which could have resulted in selection bias. Moreover, the diagnosis was made by doctors at each institution, and it was not possible to find out what additional indicators were used to determine the etiology or to rule out the possibility that some children had conductive hearing loss and that some of the children who were considered as having congenital hearing loss had experienced sudden hearing loss or that some children who were considered as having an unknown cause of hearing loss had other causes. We could not fully differentiate between the being congenital and the etiology being congenital. However, these institutions are staffed by board-certified specialists, which could lend greater credibility to the diagnosis. This survey used standard 3 frequencies to assess the hearing level, which poses limitations in understanding hearing loss configuration and how the hearing loss impacts important speech information. Another limitation lies in the time when the audiological data was obtained. Some patients had progressive hearing loss and other patients achieved hearing recovery. The survey could not obtain the time course of each patient’s hearing level. Furthermore, we cannot ignore the fact that patients who had been already diagnosed were included in our study, because the data were collected when the patients visited the institutions for the first time, and we did not ascertain whether they had consulted other institutions before presentation. However, our study is the first national survey of UHL to include a large number of participants and offers reliable data about the etiology and device use in UHL.

## 5. Conclusions

We performed a national survey of UHL in children and adults. The age distribution and the severity of each disease observed in this study were apparently not different from those reported previously. As to treatment modalities, however, the ratio of hearing aid usage or CI was different from other studies, which we consider due to Japanese government policy. Newborn hearing screening seems effective for identifying UHL in children, and hearing aid use and CI are limited in children and adults. Many patients with severe to profound UHL are far from benefiting from interventions in Japan and require further interventions such as hearing aids and CI.

## Data Availability

The data that support the findings of this study are available on request from the corresponding author, T.Y.

## Supplementary Materials

The following supporting information can be downloaded at: https://www.mdpi.com/article/10.3390/jcm12134290/s1, Table S1: items of the questionnaire (for the institutions with otolaryngology residency program).
