# Supplementary material for: Etiology, Severity, Audiogram Type, and Device Usage in Patients with Unilateral Moderate to Profound Sensorineural Hearing Loss in Japan
Source: J Clin Med. 2023 Jun 26;12(13):4290. doi: 10.3390/jcm12134290 (PMC10342665; doi:10.3390/jcm12134290)
Supplement: Supplementary file 1 [file jcm-12-04290-s001.zip › jcm-2419677-supplementary.pdf]

Table S1. Items of questionnaire（for the institutions with otolaryngology residency program）

1． Name of the institutions

2． Name of the provider, address of the provider

3． Criteria of the patients

(1) The patients who were referred to your institutions between April 2018 to March 2020 for the first time.

(2) The patients with moderate, severe, or profound sensorineural hearing loss (≥ 40dB HL) in one ear and normal hearing (< 20dB HL or appropriate for age) in the other ear. Patients with conductive hearing loss should be excluded.

4． Information about the patients

(1) ID for the study

(2) Age

(3) Gender

(4) Etiology (Chose only one choice)

① Acquired

1. Sudden Sensorineural Hearing Loss
2. Auditory tumor
3. Meniere's disease
4. Perilymphatic fistula
5. Acoustic trauma
6. Other trauma
7. Mumps
8. Meningitis
9. Other detected causes
10. Unknown

② Congenital

1. Anomaly
2. Cochlear Nerve Canal Stenosis
3. Cytomegalovirus infection
4. Other infection
5. Other detected causes
6. Unknown

(5) Severity (Chose only one choice)

① Moderate: pure-tone average at 3 frequencies (0.5Hz, 1kHz, and 2kHz) ≥ 40dB and <70 dB

② Severe: pure-tone average at 3 frequencies (0.5Hz, 1kHz, and 2kHz) ≥ 70dB and <90 dB

③ Profound: pure-tone average at 3 frequencies (0.5Hz, 1kHz, and 2kHz) ≥ 90 dB

(6) Intervention

① No

② Yes

1. Bone Anchored Hearing Aid
2. Hearing Aid
   (1) Air conductive
   (2) Bone conductive
   (3) Cartilage conductive
   (4) Contralateral routing system
3. Cochlear Implant

Items of questionnaire（for the institutions with Japan Auditory Society board members）

1．Name of the institutions

2．Name of the provider, address of the provider

3．Criteria of the patients

(3) The patients who were referred to your institutions between April 2017 to March 2020 for the first time.

(4) The patients with moderate, severe or profound sensorineural hearing loss (≥ 40dB HL) in one ear and normal hearing (< 20dB HL or appropriate for age) in the other ear. Patients with conductive hearing loss should be excluded.

4．Information about the patients

(1) ID for the study

(2) Age

(3) Gender

(4) Etiology (Chose only one choice)

① Acquired

1. Sudden Sensorineural Hearing Loss
2. Auditory tumor
3. Meniere's disease
4. Perilymphatic fistula
5. Acoustic trauma
6. Other trauma
7. Mumps
8. Meningitis
9. Other detected causes
10. Unknown

② Congenital

1. Anomaly
2. Cochlear Nerve Canal Stenosis
3. Cytomegalovirus infection
4. Other infection
5. Other detected causes
6. Unknown

(5) Pure tone audiogram data

(6) Intervention

① No

② Yes

1. Bone Anchored Hearing Aid
2. Hearing Aid
   (1) Air conductive
   (2) Bone conductive
   (3) Cartilage conductive
   (4) Contralateral routing system
3. Cochlear Implant
